# Bangladeshi crops leaf disease detection using YOLOv8

**DOI:** 10.1016/j.heliyon.2024.e36694

**Published:** 2024-09-04

**Authors:** Md. Shahriar Zaman Abid, Busrat Jahan, Abdullah Al Mamun, Md. Jakir Hossen, Shazzad Hossain Mazumder

**Affiliations:** a Department of Computer Science and Engineering, Feni University, Feni, Bangladesh; b School of Information and Communication, Griffith University, Brisbane, Australia; c Faculty of Engineering and Technology, Multimedia University, Melaka, Malaysia; d Department of Computer Science and Engineering, University of Chittagong, Chittagong, Bangladesh

**Keywords:** Technology in agriculture, Machine learning, Object detection, Image classification, Crop leaf disease detection, CNN, YOLOv8

## Abstract

The agricultural sector in Bangladesh is a cornerstone of the nation's economy, with key crops such as rice, corn, wheat, potato, and tomato playing vital roles. However, these crops are highly vulnerable to various leaf diseases, which pose significant threats to crop yields and food security if not promptly addressed. Consequently, there is an urgent need for an automated system that can accurately identify and categorize leaf diseases, enabling early intervention and management. This study explores the efficacy of the latest state-of-the-art object detection model, YOLOv8 (You Only Look Once), in surpassing previous models for the automated detection and categorization of leaf diseases in these five major crops. By leveraging modern computer vision techniques, the goal is to enhance the efficiency of disease detection and management. A dataset comprising 19 classes, each with 150 images, totaling 2850 images, was meticulously curated and annotated for training and evaluation. The YOLOv8 framework, known for its capability to detect multiple objects simultaneously, was employed to train a deep neural network. The system's performance was evaluated using standard metrics such as mean Average Precision (mAP) and F1 score. The findings demonstrate that the YOLOv8 framework successfully identifies leaf diseases, achieving a high mAP of 98% and an F1 score of 97%. These results underscore the significant potential of this approach to enhance crop disease management, thereby improving food security and promoting agricultural sustainability in Bangladesh.

## 1 Introduction

Bangladesh is one of the world's top five rice producing and consuming countries. As an agricultural country, about 135 million people in Bangladesh consider rice as their staple food [1] and making it the third largest rice producer in the world [2]. Rice production of Bangladesh increased from 15.1 million tons in 1972 to 56.9 million tons in 2021 growing at an average annual rate of 2.89% [3]. Also, corn is a significant crop for farmers in the country. During the current financial year 2022-23, farmers have grown the grain on more than 14 lakh acres, showed estimates by the Department of Agricultural Extension (DAE) [4]. In 2020, corn production for Bangladesh was 4,700 thousand tons. Between 2011 and 2020, corn production of Bangladesh grew substantially from 1,954 to 4,700 thousand tons rising at an increasing annual rate that reached a maximum of 17.14% in 2019 and then decreased to 14.63% in 2020 [5]. If the northern part of the country is harvesting corn, then south region of the country is busy cultivating wheat. In 2020, wheat production for Bangladesh was 1,180 thousand tons. Between 1999 and 2020, wheat production in Bangladesh was decreasing on average by 0.44% each year, although before that, it grew from 111 thousand tons in 1973 to 1,988 thousand tons in 1999 [6]. Potato is one of the main food crops in Bangladesh after rice, corn and wheat. In 2021, Bangladesh exported $14.3M in Potatoes, making it the 35th largest exporter of Potatoes in the world. At the same year, Potatoes was the 98th most exported product in Bangladesh. The main destination of Potatoes exports from Bangladesh are: Malaysia ($7.42M), Sri Lanka ($3.87M), Nepal ($1.1M), Singapore ($667k), and United Arab Emirates ($624k) [7]. Tomato is one of the most important and popular vegetables in Bangladesh. In 2021, Bangladesh exported $89.8k in Tomatoes, making it the 92nd largest exporter of Tomatoes in the world. At the same year, Tomatoes was the 491st most exported product in Bangladesh. The main destination of Tomatoes exports from Bangladesh are: Malaysia ($88.6k), Singapore ($532), United Arab Emirates ($402), and Maldives ($227) [8]. So therefore, we chose these five crops because they play an important role in the economy of Bangladesh. We have worked with the updated version YOLOv8 of the Convolutional Neural Network (CNN). Convolutional Neural Network (CNN) is one of the among best techniques used in deep learning. It has been enthusiastically used for solving computer based issues including picture classification, object segmentation, image analysis, etc. YOLO is an algorithm of object detection that partitions images into a grid structure. Every grid cell has the responsibility of identifying objects. As a result, we decided to convey the current work using the YOLOv8. As far as we are aware, no study has used YOLOv8 to detect disease on Rice, Corn, Wheat, Potato and Tomato leaf diseases, as is done in the current study. Hence, the goal of this study was to pinpoint disease from the captured images using the YOLOv8. The effects of leaf diseases on the agriculture sector in Bangladesh are significant and far reaching. As a country heavily dependent on agriculture for its economy and food security, any impact on crop health can have profound consequences for farmers, the agricultural industry, and the overall economy. Leaf diseases pose many challenges that adversely affect crop production, quality, and profitability. We used several types of diseases of rice, corn, wheat, potato and tomato leaf in this work.

The use of CNNs and object detection models like YOLO has not been limited to agriculture. In medical imaging, these models have shown remarkable success in diagnosing diseases from medical scans and images. For example, CNNs have been used to detect pneumonia from chest X-rays, identify tumors in MRI scans, and classify skin lesions in dermatology. These applications underscore the versatility and effectiveness of deep learning models in various fields, highlighting their potential for improving disease diagnosis and management in both agriculture and medicine. By employing YOLOv8, this study aims to bring similar advancements to the agricultural sector in Bangladesh, enhancing crop disease management and contributing to overall agricultural sustainability. Though there are some studies that develop models that can classify affected crop leaves but these are hard to deploy in real-life scenario. In our research, we developed a pipeline to train the YOLOv8 based pre-trained model that can detect disease in crop leaves effectively and can also be easily deployed to end users through mobile or web apps.

## 2 Literature study

Inception ResNet v2 is a convolutional neural network (CNN) model, employed with a transfer learning strategy to recognize diseases in images of rice leaves. This model is optimized for the classification task and obtained. Three major rice leaf diseases are leaf Blast, Bacterial Blight, and Brown spot. The accuracy of this model is 95.67% [9].

In this paper, The CNN architecture is VGG16 based and has been trained and tested using a dataset compiled from both rice field samples and online sources. The accuracy of the proposed model is 92.46%. There are 1649 images depicting diseased rice leaves, showcasing the three prevalent diseases: Brown spot, Rice leaf blast and Bacterial leaf blight. [10].

This study aimed to create an application for the detection of leaf blast or brown spot disease on rice leaves. Two hundred rice leaf images of a custom dataset trained by YOLO Algorithm. Their findings revealed that the device achieved an accuracy rate of 90.00% for leaf blast disease and 70.00% for class 2 brown spot diseases [11].

This study used lightweight Artificial Intelligence techniques and computer concepts for a system for detecting diseases in rice leaves. From 300 photos, they identified 3 diseases affecting rice plants: Hispa, Brown Spot and Leaf Blast. This paper's accuracy achieved 97.50%. [12].

Image processing techniques were applied in conjunction with Support Vector Machine (SVM) to detect diseases in rice. This approach achieved an impressive accuracy of 97.2%. The process involved extracting shape and texture features after isolating the affected areas within the images. Furthermore, SVM was employed to classify diseases such as Bacterial leaf blight, rice sheath blight, and rice blast disease [13].

A neural network model utilizing YOLO was employed to categorize 5 types of herb leaves: Basil, Betel, Aloe Vera, Mehndi and Mint. The model achieved an impressive classification accuracy of approximately 95% [14].

In this paper, a proficient technique for identifying face masks is presented, employing a deep learning model based on “YOLOv5”. The algorithm undergoes training across five different numbers of epochs. Among the 86 tested images, the deep learning model trained for 300 epochs exhibits the highest performance, achieving an accuracy of 96.5% along with the best precision and recall [15].

In this paper, Food photos with mold growing on their surfaces were made using a collection of 2050 photographs. They were found in their laboratory (850 images) and on the internet (1200 images). The YOLOv5 pre trained method was used to train the dataset. The current YOLOv5 model outperformed YOLOv3 and YOLOv4 in terms of precision, recall, and average precision (AP) [16].

This study focuses on three common diseases affecting apple trees: black rot, fish scale disease, and snow apple rust. To tackle this, they utilized the YOLOv5 model, which is based on deep learning techniques, and incorporated a novel stable information derived from an autoencoder. Their training process involved the use of a PlantVillage dataset, comprising 5740 images, and was conducted on Google Colab. The dataset was divided into training, validation, and testing sets, with 70% used for training (4018 photos), 20% for validation (1148 images), and 10% for testing (574 images). After completing their training, they achieved a detection rate of 81.28% and a classification rate of 91.93% when employing the PlantVillage dataset [17].

For the agricultural crop protection system to operate effectively, it is crucial to precisely diagnose and categorize plant diseases. Initially, visual data was collected, and this image data was subsequently organized into three distinct disease categories: Leaf Blast, Brown Spot, and Healthy Leaf. The dataset comprises a total of 3355 images, encompassing both healthy and diseased rice leaves. Its superior performance over Inception v3 is attributed to more efficient utilization of model parameters. This classification process enables accurate diagnosis and treatment of the various diseases that affect rice plants. This study primarily focused on identifying different diseases that impact various rice varieties [18].

The automated leaf disease diagnosis system serves as a precision agriculture solution, employing Computer Vision, Image Processing, and Machine Learning algorithms to forecast diseases by examining images of affected leaves. Previously, farmers were required to dispatch diseased leaves to pathology labs for disease confirmation, a procedure that consumed considerable time. This article introduces a machine learning based framework for the categorization and identification of leaf diseases, presenting a more streamlined and efficient approach [19].

Deep learning stands as a prominent field of study in image processing and computer vision, well regarded for its exceptional performance in diverse domains. Nevertheless, the utilization of deep learning methods for plant disease recognition has been somewhat constrained. This article presents a potent and efficient network structure, termed MobIncNet, designed in the context of recognition and identification of crop diseases. Empirical findings reveal that this approach attains remarkable outcomes, with a mean recognition accuracy of 99.21% from a publicly accessible dataset and 97.89% on a dataset gathered from a particular local source [20].

The YOLOv4 framework, grounded in Convolutional Neural Network technology, was utilized for real time object detection. They harnessed a distinctive model, YOLOv4 tiny, to emphasize the identification and assessment of plant leaf diseases, providing preventive measures for each ailment. This system has been integrated with an Android application to furnish users with a user friendly interface for swift leaf disease recognition. These diseases can result from infections or living (biological) factors [21].

Rice serves as a vital cereal food crop, being the primary sustenance for half of the global population, with edible rice produced from the seeds of paddy plants. The wide spread bacterial and fungal diseases that afflict rice plants, amplified by climate change and global warming, significantly compromise the quality and yield of rice. The present study proposes an automated solution in the form of a smartphone application (E-crop doctor) for disease identification in paddy leaves and the subsequent recommendation of pesticide use to aid farmers [22].

A novel deep learning technique is proposed for classifying potato leaf diseases into five categories: Potato Late Blight, Potato Early Blight, Potato Leaf Roll, Potato Verticillium Wilt, and Healthy. Utilizing the Plant Village Dataset and additional manually gathered data, the model employs a pre-trained Efficient DenseNet with an added transition layer and reweighted cross-entropy loss function to handle class imbalance. This approach achieved a 97.2% accuracy, demonstrating superior performance in detecting and classifying potato leaf diseases compared to existing models [23].

A deep ensemble learning model (DELM) is proposed for autonomous identification of tomato plant leaf diseases. Utilizing transfer learning on pretrained models and augmentation techniques like image enhancement, rotation, and scaling, the model combats overfitting. The research evaluates single and ensemble learning models using a publicly available dataset with ten biotic disease classes. A single VGG16 model achieved 98% accuracy and a 93.25% F1-score. The ensemble of VGG16, InceptionV3, and GoogleNet outperformed other models, demonstrating superior accuracy in classifying tomato plant leaf diseases [24].

Table 1 shows comparative study on similar researches.

**Table 1:** Comparison chart of studies.

Study	Algorithm	Dataset	Diseases Identified	Accuracy (%)	Additional Notes
Inception ResNet v2 for rice leaves [9]	Inception ResNet v2	-	Leaf Blast, Bacterial Blight, Brown Spot	95.67	Transfer learning strategy
VGG16 for rice leaves [10]	VGG16	1649 images	Brown Spot, Rice Leaf Blast, Bacterial Leaf	92.46	Dataset compiled from field samples and online
YOLO for rice leaf diseases [11]	YOLO	200 images	Leaf Blast, Brown Spot	90.00 (Leaf Blast), 70.00 (Brown Spot)	Custom dataset
Lightweight AI for rice leaf diseases [12]	Lightweight AI	300 images	Hispa, Brown Spot, Leaf Blast	97.5	-
SVM for rice leaf diseases [13]	SVM	-	Bacterial Leaf Blight, Rice Sheath Blight	97.2	Image processing techniques used
YOLO for herb leaves [14]	YOLO	-	Basil, Betel, Aloe Vera, Mehndi, Mint	95	-
YOLOv5 for face mask detection [15]	YOLOv5	86 images	-	96.5	Training across five different epochs
YOLOv5 for food mold detection [16]	YOLOv5	2050 images	Mold detection	-	Outperformed YOLOv3 and YOLOv4
YOLOv5 for apple tree diseases [17]	YOLOv5	5740 images	Black Rot, Fish Scale Disease, Snow Apple	91.93 (Classification)	Training on PlantVillage dataset
Classification of rice leaf diseases [18]	-	3355 images	Leaf Blast, Brown Spot, Healthy Leaf	-	Superior performance over Inception v3
ML-based framework for leaf disease diagnosis [19]	ML	-	-	-	Precision agriculture solution
MobIncNet for crop disease recognition [20]	MobIncNet	Publicly available dataset and local dataset	Various crop diseases	99.21 (Public), 97.89 (Local)	Designed for crop disease recognition and identification
YOLOv4 tiny for plant leaf disease [21]	YOLOv4 tiny	-	-	-	Integrated with an Android application
Smartphone application for rice disease identification [22]	-	-	-	-	Automated solution with pesticide recommendations
Proposed technique for potato disease management [23]	Efficient DenseNet	‘The Plant Village’ + manually gathered data	Potato Late Blight (PLB), Potato Early Blight (PEB), Potato Leaf Roll (PLR), Potato Verticillium Wilt (PVw), Potato Healthy (PH)	97.2	First technique to detect and classify four diseases in potato leaves; utilizes reweighted cross-entropy loss function to handle imbalanced data; dense connections with regularization to minimize overfitting
Deep ensemble learning model for tomato plant leaf diseases [24]	DELM (VGG16, InceptionV3, GoogleNet)	Publicly available dataset with ten different biotic disease classes	Various tomato plant leaf diseases	98% (VGG16), higher for ensemble	Utilizes transfer learning and augmentation techniques to combat overfitting; ensemble model shows more accurate results than single models

While numerous studies have applied various machine learning and deep learning techniques for crop leaf disease detection, many have relied on earlier versions of object detection models such as YOLOv3 or Faster R-CNN, which, although effective, often present limitations in terms of speed, accuracy, and the ability to generalize across diverse datasets. A significant gap identified in the existing literature is the need for a model that not only achieves high accuracy in disease detection but also operates efficiently in real-time, making it feasible for practical, large scale agricultural applications. YOLOv8, the latest iteration in the YOLO series, addresses these challenges by incorporating advanced neural network architectures and improved training methodologies that enhance both speed and accuracy. Its ability to perform well on complex and varied datasets, including those with small or overlapping objects, makes it particularly well-suited for the intricate patterns found in crop leaf diseases. Therefore, YOLOv8 was chosen for this study to leverage its state-of-the-art performance, ensuring robust and real-time detection of leaf diseases in Bangladeshi crops, which is critical for timely intervention and management in agriculture.

## 3 Materials and methods

**Figure 1:**
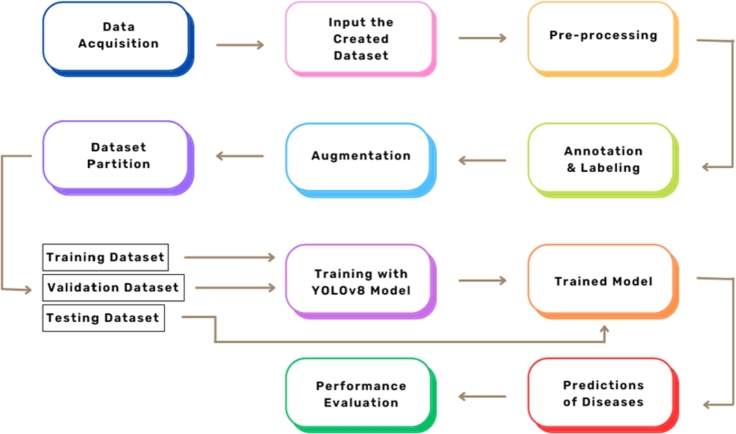
Flow Diagram of Proposed System.

The methodology involves several key stages. First, data acquisition is conducted to gather relevant datasets. The created dataset is then input and pre-processed, followed by annotation and labeling. Augmentation techniques are applied to enhance the dataset before partitioning it into training, validation, and testing sets. The YOLOv8 model is trained using the training dataset. The trained model is then used for predicting diseases. Performance evaluation is carried out to assess the model's accuracy. This iterative process ensures continuous improvement through feedback loops, refining both the dataset and the model's performance. Fig. 1 illustrates the flow diagram of proposed system.

### 3.1 Data acquisition & creating dataset

We sourced images of both diseased and healthy crop leaves from the Plant Village dataset from Kaggle, totaling over 21,000 images. From this collection, we specifically selected 150 images for each category. The dataset comprises of 19 classes that are divided into five groups: rice, corn, wheat, potato, and tomato. Within the rice group, there are three classes: brown spot, leaf blast, and healthy. The potato group encompasses three classes: early blight, late blight, and healthy. In the corn group, you'll find four classes: common rust, gray leaf spot, northern leaf blight, and healthy. The wheat group includes three classes: brown rust, yellow rust, and healthy. Lastly, the tomato group features six classes: bacterial spot, late blight, early blight, leaf mold, curl virus, and healthy.

The primary objective of selecting of 150 images per class, was to create a balanced and manageable dataset that accurately represents the various classes of leaf diseases prevalent in Bangladeshi crops. Ensuring that each class has an equal number of images helps prevent bias in the model training process, thereby enhancing the reliability of the detection results. Making the most of our available resources, our dataset with 150 images per class strikes a practical balance between dataset size and the feasibility of extensive model training and validation. Larger datasets, while potentially offering more variety, would require significantly more resources for processing and model optimization, which might not be feasible within the scope of this study. Moreover, previous studies have shown that modern object detection models like YOLOv8 can achieve robust performance with relatively smaller, well-curated datasets when coupled with effective data augmentation techniques. Data augmentation was utilized in this study to artificially increase the variety and volume of training data, thus mitigating the limitations imposed by the smaller dataset size.

Table 2 shows the list of selected diseases for each crop along with their respective name and image.

**Table 2:**
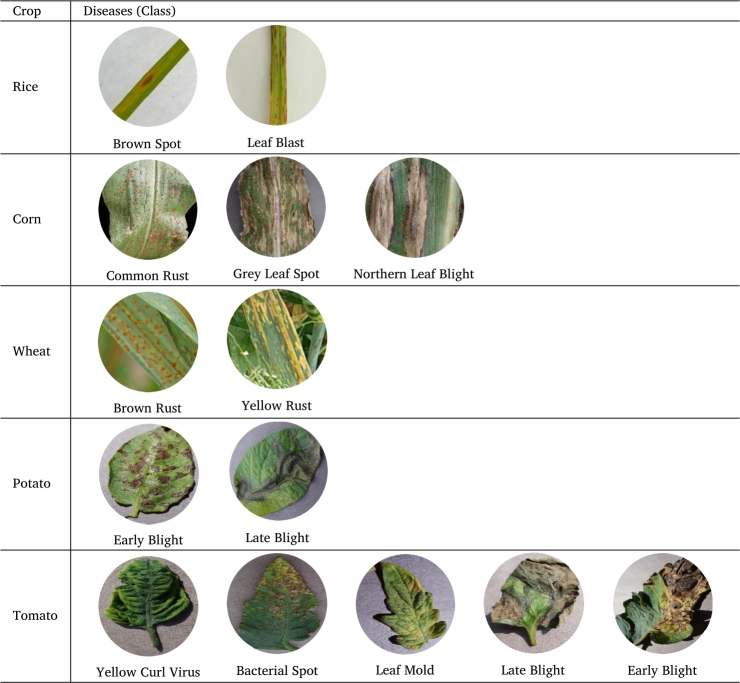
List of crop diseases.

### 3.2 Data preprocessing

**Figure 2:**
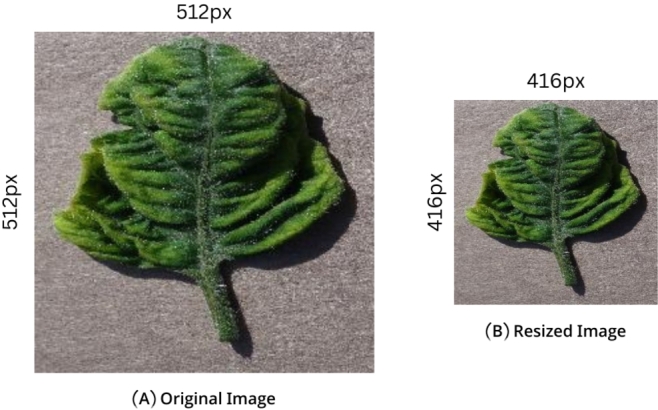
(A, B). Image Resizing.

**Figure 3:**
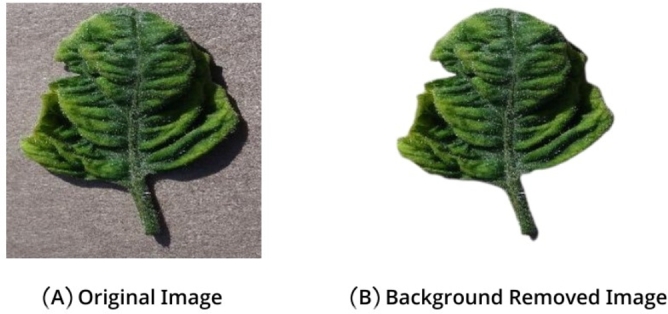
(A, B). Background Removing.

In the plant Village dataset, there were images of different sizes. So, we resized the selected images into (416 x 416) px. After that, we removed the background from each image. Shown in Fig. 2 (A, B) & 3 (A, B)

### 3.3 Data annotation & labeling

**Figure 4:**
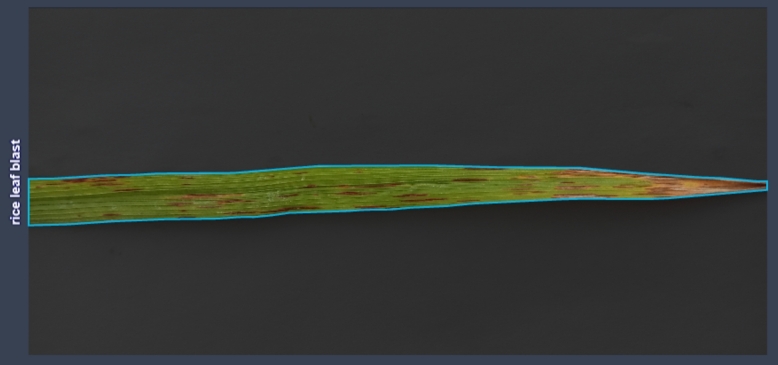
Data Annotation & Labeling.

Data annotation is the process of labeling and tagging the collected images to identify and highlight the regions of interest. We used the Roboflow platform for annotation because this platform annotates data in YOLO format. Roboflow facilitated this task by providing an intuitive interface and a range of annotation tools that allowed us to annotate each image with bounding boxes around the diseased regions. The annotation process involved carefully examining each image and identifying the specific crops and disease classes. With Roboflow's assistance, we could accurately annotate the dataset with the corresponding classes, ensuring that our model could distinguish and detect the different leaf diseases effectively in Fig. 4.

### 3.4 Data augmentation

**Figure 5:**
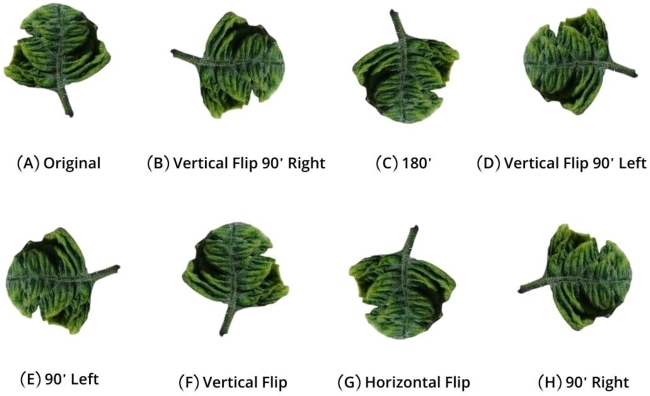
(A - H). Data Augmentation.

Following the image annotation process, we employed Roboflow for further augmentation. The purpose of applying data augmentation was to increase image count and introduce dataset diversity, a strategy commonly used to mitigate overfitting problems in smaller datasets. The Fig. 5 (A – H) depicts the utilization of augmentation methods, encompassing rotation (clockwise & anti-clockwise) and flip (horizontal and vertical), to create new images based on the original dataset. As a result of this random augmentation, our dataset now contains 5750 images, an increase from the initial 2850 images.

### 3.5 Dataset partition

**Figure 6:**
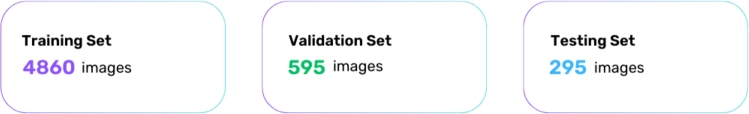
Dataset Partition.

Our dataset now includes 5750 images, organized into three separate subsets: the training set, validation set, and testing set. As shown in Fig. 6, this distribution allocates 70% of the images to the training set, 20% to the validation set, and 10% to the testing set

### 3.6 YOLOv8 architecture

**Figure 7:**
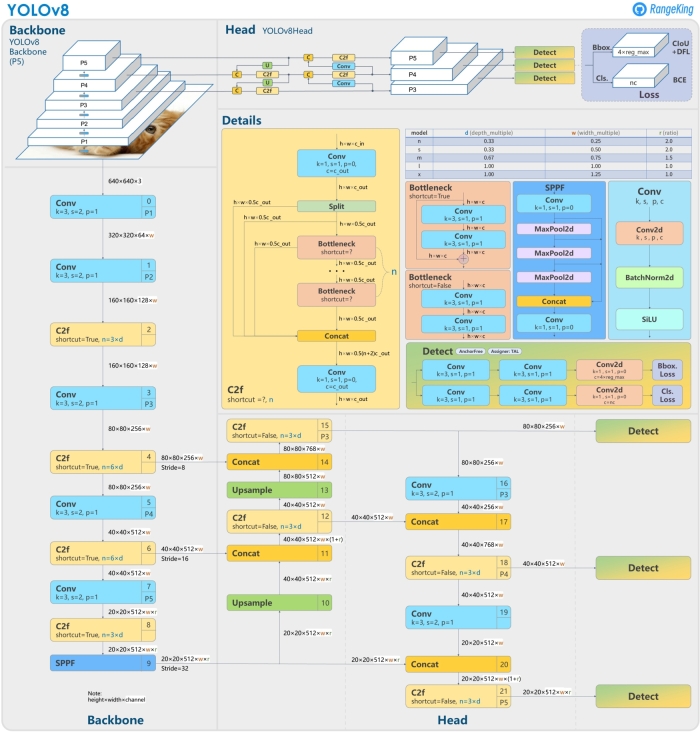
YOLOv8 Architecture. [25].

As shown in Fig. 7, the Architecture of YOLOv8 is divided into two main parts – the backbone and the head

1. Backbone: The primary function of the Backbone is to extract crucial features from an input image. In YOLOv8, CSP (Cross Stage Partial Networks) is employed as the backbone to extract valuable and diverse features from the input image. The BottleneckCSP module is chiefly responsible for performing feature extraction on the feature map, enabling the extraction of rich information from the image. When compared to other extensive convolutional neural networks, the structure of BottleneckCSP effectively minimizes the redundancy by utilizing gradient information during the optimization process of convolutional neural networks. It constitutes a substantial portion of the entire network's parameter quantity. Meanwhile, the SPP (Spatial Pyramid Pooling) module focuses on expanding the network's receptive field and capturing features of various scales. The YOLOv8 backbone is based on the CSPDarknet53 architecture, which is a modified version of the Darknet architecture.
2. Head: The primary role of the Head is to oversee the final step of detection. It leverages anchor boxes to create the ultimate output vectors, encompassing class probabilities, object absence scores, and bounding box information. The head is a CNN that takes the features extracted by the backbone and produces the final output of the model, which is a set of bounding boxes and class probabilities for each object in the image.

YOLOv8 builds on the previous versions of YOLO by introducing a number of architectural improvements, including:

• Spatial attention: YOLOv8 uses a spatial attention module to focus on the most relevant parts of the image for each object detection task. This helps to improve the accuracy and robustness of the model.
• Feature fusion: YOLOv8 fuses features from different layers of the CNN to create richer feature maps. This also helps to improve the accuracy and robustness of the model.
• Context aggregation: YOLOv8 uses a context aggregation module to aggregate contextual information from different parts of the image. This helps to improve the model's ability to detect objects in complex and challenging scenes.

### 3.7 Training with YOLOv8 model & prediction results

**Figure 8:**
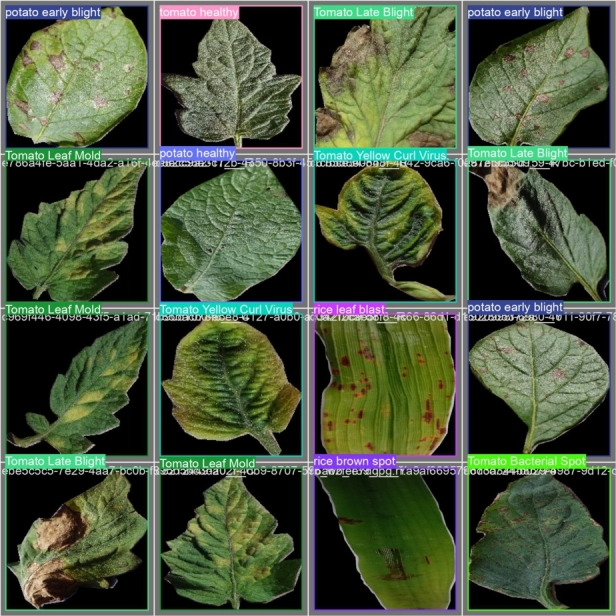
Images that were given to test.

**Figure 9:**
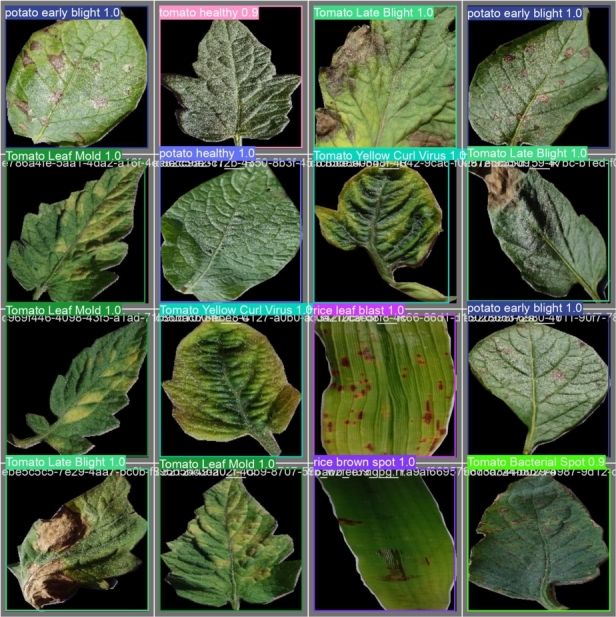
Prediction Results of Tested Images.

We used a Roboflow custom notebook that is based on YOLOv8 so that we could train our model in a customized way. The model is created in PyTorch and runs on the CPU and GPU. We run the custom notebook in Google Colab. We installed YOLOv8 from pip. Then we imported our annotated dataset by using Roboflow generated URL. We trained our model on 100 epochs, and the batch size was 16 per epoch, which took over 3 hours to complete. In the YOLOv8 directory, we can find images of testing results and graphs of the performance of our trained model. In Figure 8, Figure 9, We provided the results of the testing set.

## 4 Results and discussion

The model improves the detection of crop leaf diseases as a YOLOv8 feature. The model's precision, recall, mAP, and F1 score increased. Additionally, it was shown that performance was better trained when the image quality is good.

### 4.1 Performance evaluation

Equation (1) provides the ratio of correctly predicted positive observations to the total predicted positives. It is a measure of the accuracy of the positive predictions. Equation (2) provides the ratio of correctly predicted positive observations to the all observations in actual class. It is a measure of the ability of the classifier to capture all the positive instances. Equation (3) provides insight into the model's test accuracy, which is an additional consideration. The F1 score, ranging from 0 (representing zero precision or zero recall) to 1 (indicating perfect precision and recall), is used to gauge performance. Additionally, mean average precision (mAP), as defined in Equation (4), is calculated by taking the average of the precision values (AP) across all classes. mAP serves as a performance metric for machine learning algorithms. In the context of the challenge of identifying suitable emergency landing spots, “True Positive” signifies the count of correctly identified valid locations as suitable for landing by the algorithm. “False positive” denotes the number of incorrect identifications of unsuitable landing spots as suitable, while “false negative” represents the instances where appropriate landing spots were missed.(1)
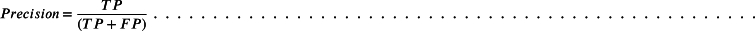
(2)
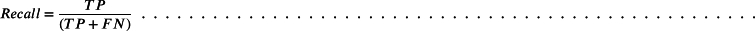
(3)
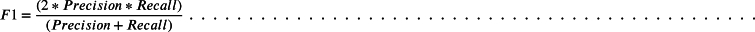
(4)
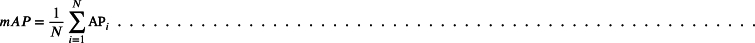


Here, *TP* = True Positive, *FP* = False Positive, *FN* = False Negative, *AP* = Average Precision, *mAP* = Mean Average Precision, *N* = Number of Classes.

### 4.2 F1 confidence curve

**Figure 10:**
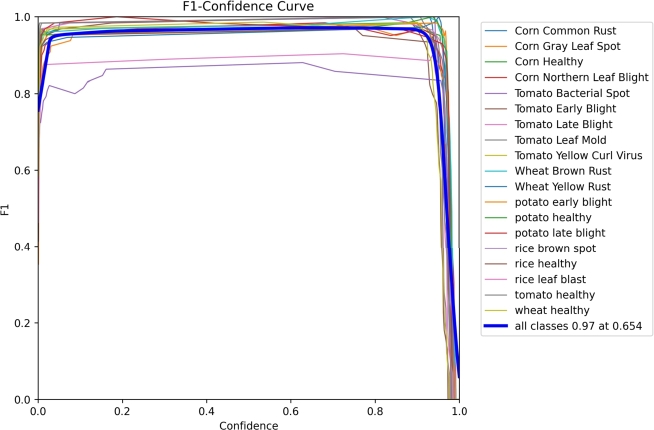
F1 Graph.

The F1 measure computes the balanced harmonic mean of a classifier's precision (P) and recall (R), resulting in the F1 score. This indicates that both metrics carry equal importance. The graph displays a confidence value of 0.654, which aligns with the highest F1 score of 0.97, effectively balancing precision and recall. As depicted in Fig. 10, a higher confidence value and F1 score are typically preferred.

### 4.3 Precision confidence curve

**Figure 11:**
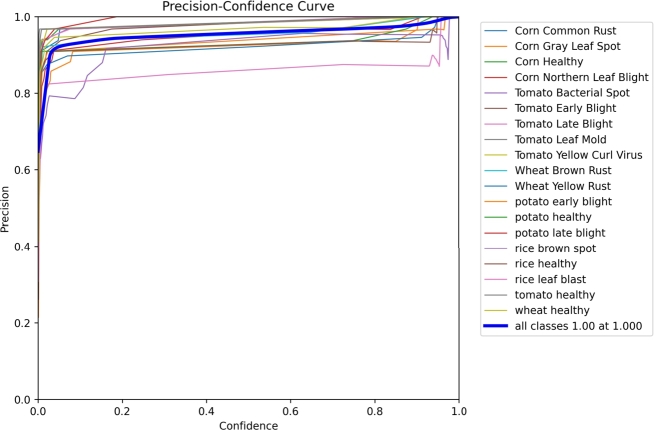
Precision Graph.

The accuracy of our estimation improves as the sample size increases, and the confidence interval illustrates the precision of our ability to describe the effect size, with sample size often being a critical factor in determining this precision. The connection between precision and the confidence interval is evident. Fig. 11 shows that precision values of 1.00 fall within the 1.000 confidence interval for an effect.

### 4.4 Recall confidence curve

**Figure 12:**
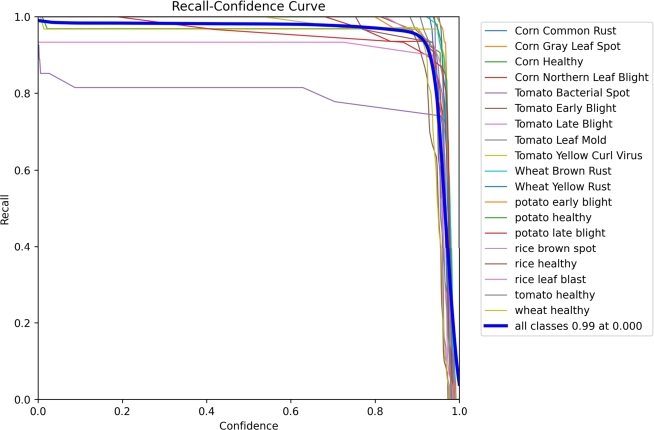
Recall Graph.

An increased sample size results in a more accurate estimation, and the confidence interval serves as an indicator of our ability to precisely convey the magnitude of the effect. Frequently, the sample size is a pivotal determinant of this accuracy. The alignment between the accuracy measure and the confidence interval is apparent (as seen in Fig. 12). The recall values of 0.000 fall within the 0.99 confidence interval for the effect.

### 4.5 Precision recall curve

**Figure 13:**
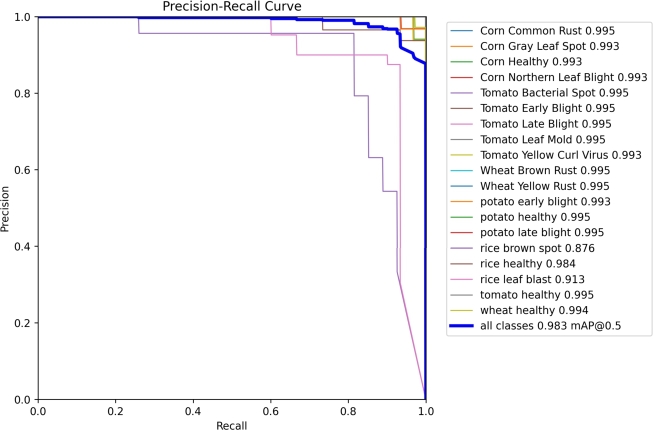
Precision Recall Graph.

The precision recall curve illustrates the tradeoff between precision and recall at different thresholds. Elevated precision corresponds to a reduced false positive rate, whereas increased recall is tied to a decreased false negative rate. A substantial area under the curve indicates a combination of elevated recall and precision. As depicted in Fig. 13, we achieved a Mean Average Precision (mAP) of 0.983 using the precision recall curve.

### 4.6 Loss analysis

**Figure 14:**
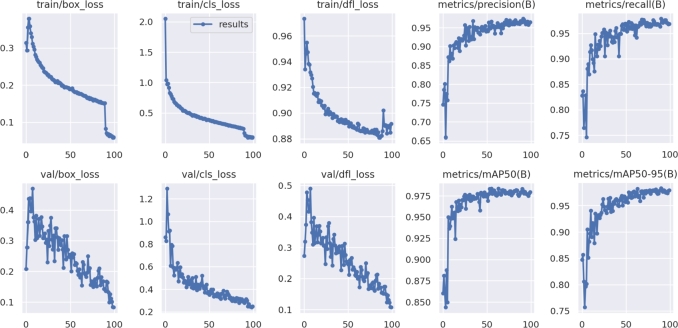
Loss Graphs.

The network underwent training using the combined training and validation sets. Fig. 14 displays the curves depicting the values of the loss functions for the training and validation sets, encompassing the detection frame loss, detection object loss, and classification loss.

### 4.7 Model accuracy comparison

**Figure 15:**
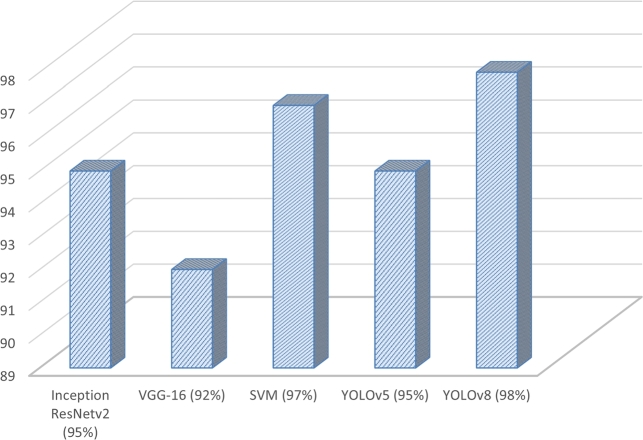
Model Accuracy Comparison Chart.

The achieved outcomes showcase the efficacy of the YOLOv8 model, showcasing an outstanding mean average precision (mAP) of 98% and an impressive F1 score of 97%. From the below chart (Fig. 15) we can see that YOLOv8 outperformed previous models.

## 5 Conclusion

The primary goal of our research is to advance agricultural technology by improving production efficiency and simplifying disease detection processes. This technology offers substantial benefits to farmers, including time savings and more effective disease management. For this study, a dataset encompassing 19 categories, each with 150 images, totaling 2850 images, was meticulously curated and annotated for both training and evaluation purposes. Utilizing the YOLOv8 architecture, we trained a deep neural network capable of detecting multiple objects simultaneously. Performance metrics such as mean Average Precision (mAP) and F1 score were used to evaluate the system. The results highlight the robustness of our approach, with an impressive mAP of 98% and an F1 score of 97%. This study confirms the successful application of the YOLOv8 framework in identifying leaf diseases across five major crops in Bangladesh. The exceptional performance metrics underscore the potential of this approach to significantly aid in crop disease management, thereby contributing to food security and the sustainability of agriculture. Our work offers a substantial contribution to the field of agricultural technology, providing a scalable solution for early disease detection and effective intervention strategies in crop management.

## Funding statement

The authors received no specific funding for this study.

## CRediT authorship contribution statement

**Md. Shahriar Zaman Abid:** Writing – original draft, Visualization, Validation, Software, Methodology. **Busrat Jahan:** Writing – original draft, Supervision, Investigation, Formal analysis, Conceptualization. **Abdullah Al Mamun:** Writing – review & editing, Supervision, Investigation, Funding acquisition. **Md. Jakir Hossen:** Writing – review & editing, Validation, Project administration, Funding acquisition.

## Declaration of Competing Interest

The authors declare the following financial interests/personal relationships which may be considered as potential competing interests: Dr. Md. Jakir Hossen reports financial support was provided by 10.13039/100012024Multimedia University. Dr. Md. Jakir Hossen reports a relationship with Multimedia University that includes: employment and funding grants. Dr. Md. Jakir Hossen has patent pending to Patent is not applied yet. No conflict of interest If there are other authors, they declare that they have no known competing financial interests or personal relationships that could have appeared to influence the work reported in this paper.

## Data Availability

The data that support the findings of this study are not publicly available. Data will be made available on request. Please contact the corresponding author to request the data.
